# Supplementary material for: Cost-Effectiveness of Glucosamine in Osteoarthritis Treatment: A Systematic Review
Source: Healthcare (Basel). 2023 Aug 18;11(16):2340. doi: 10.3390/healthcare11162340 (PMC10454215; doi:10.3390/healthcare11162340)
Supplement: Supplementary file 1 [file healthcare-11-02340-s001.zip › healthcare-2553294-supplementary.pdf]

Table S1. Quality assessment of selected reports based on QHES Instrument 1

Question	Bruyère et al., 2019	Scholtissen et al., 2010	Segal et al., 2004	Black et al., 2009	Bruyère et al., 2021	Luksameesate et al., 2022	Bruyère et al., 2023
Was the study objective in a clear, specific, and measurable manner?	Yes	Yes	Yes	Yes	Yes	Yes	Yes
Were the perspective of the analysis (societal, third-party payer, etc.) and reasons for its selection stated?	No	No	No	No	No	Yes	No
Were variable estimates used in the analysis from the best available source (i.e., randomized control trial - best, expert opinion - worst)?	Yes	Yes	Yes	Yes	Yes	Yes	Yes
If estimates came from a subgroup analysis, were the groups prespecified at the beginning of the study?	No	No	No	No	No	No	No
Was uncertainty handled by (1) statistical analysis to address random events, (2) sensitivity analysis to cover a range of assumptions?	Yes	Yes	Yes	Yes	Yes	Yes	Yes
Was incremental analysis performed between alternatives for resources and costs?	Yes	Yes	Yes	Yes	Yes	Yes	Yes
Was the methodology for data abstraction (including the value of health states and other benefits) stated?	Yes	Yes	Yes	Yes	Yes	Yes	Yes
Did the analytic horizon allow time for all relevant and important outcomes? Were benefits and costs that went beyond 1 year discounted (3% to 5%) and justification given for the discount rate?	No	No	Yes	Yes	No	No	No
Was the measurement of costs appropriate and the methodology for the estimation of quantities and unit costs clearly described?	Yes	Yes	Yes	Yes	Yes	Yes	Yes

Were the primary outcome measure(s) for the economic evaluation clearly stated and did they include the major short-term was justification given for the measures/scales used?	Yes	Yes	Yes	Yes	Yes	Yes	Yes
Were the health outcomes measures/scales valid and reliable? If previously tested valid and reliable measures were not available, was justification given for the measures/scales used?	Yes	Yes	Yes	Yes	Yes	Yes	Yes
Were the economic model (including structure), study methods and analysis, and the components of the numerator and denominator displayed in a clear, transparent manner?	Yes	Yes	Yes	Yes	Yes	Yes	Yes
Were the choice of economic model, main assumptions, and limitations of the study stated and justified?	Yes	Yes	Yes	Yes	Yes	Yes	Yes
Did the author(s) explicitly discuss direction and magnitude of potential biases?	Yes	Yes	Yes	Yes	Yes	Yes	Yes
Were the conclusions/recommendations of the study justified and based on the study results?	Yes	Yes	Yes	Yes	Yes	Yes	Yes
Was there a statement disclosing the source of funding for the study?	Yes	Yes	Yes	Yes	Yes	Yes	Yes
**TOTAL**	88	88	95	95	88	92	88
**QUALITY**	High	High	High	High	High	High	High

2
